# Characterization of the complete chloroplast genome of *Trachycarpus fortunei*

**DOI:** 10.1080/23802359.2020.1800432

**Published:** 2020-07-31

**Authors:** Xiao Feng, Zhao Yang, Wang Xiu-Rong, Tian Hong Hong

**Affiliations:** a College of Forestry, Guizhou University, Guiyang, PR China; b Institute for Forest Resources & Environment of Guizhou, Guizhou University, Guiyang, PR China;; c Key Laboratory of Forest Cultivation in Plateau Mountain of Guizhou Province, Guizhou University, Guiyang, PR China; d Key Laboratory of Plant Resource Conservation and Germplasm Innovation in Mountainous Region (Ministry of Education), Guizhou University, Guiyang, PR China

**Keywords:** *Trachycarpus fortunei*, chloroplast genome, phylogenetic analysis

## Abstract

*Trachycarpus fortunei* (Hook.) H. Wendl. (Fam.: Palmae; Gen.: *Trachycarpus*) is an evergreen tree that is widely distributed in China. In this study, *T. fortunei* complete chloroplast (cp) genome was assembled. The total cp genome size of *T. fortunei* was 158,613 bp in length, containing a large single-copy region of 86,422 bp, a small single-copy region of 17,847 bp, and a pair of inverted repeat regions of 27,172 bp. The overall GC-content of *T. fortunei* cp genome was 37.21%. It encodes a total of 109 unique genes, including 79 protein-coding genes, 26 *tRNA* genes, four *rRNA* genes. Twelve genes contain a single intron and 11 genes have two introns. Phylogenetic analysis results reveal that *T. fortunei* was closely related to *Chamaerops humilis*.

*Trachycarpus fortunei* (Hook.) H. Wendl. (Fam.: Palmae; Gen.: *Trachycarpus*) is an evergreen tree that is widely distributed in China, where its leaf sheath fiber is often used as a rope and its unopened flower buds, also known as ‘brown fish,’ are edible and consumed (Yunfa 2005).

The cp genome contains a large amount of genetic information and has highly conservative characteristics. *Trachycarpus fortunei* seeds were collected in Guiding County, Guizhou Province, China (E: 107°07′42″, N: 26°13′19″). The seeds were germinated and nursed in the laboratory (the seed specimen is accessible at the Institute for Forest Resources & Environment of Guizhou, Guizhou University (accessions No. TF-001-2)), total genome DNA of collected annual new needles was extracted with EasyPure^®^ Plant Genomic DNA Kit (TransGen Biotech, Beijing, China). Total DNA was used to generate libraries with an average insert size of 400 bp, which were sequenced using the Illumina NovaSeq platform. *Trachycarpus fortunei* cp genome sequence was assembled by SPAdes (Bankevich et al. 2012) and A5-miseq (Coil et al. 2015) fragment assembly. The complete cp genome of *T. fortunei* was annotated in CPGAVAS2 (http://47.96.249.172:16019/analyzer/home, Shi et al. 2019). The annotated cp genome sequence has been deposited into the Genbank (accession number: MT712077).

*Trachycarpus fortunei* cp genome exhibited a general quadripartite structure of plants, with two reverse repeated regions (IRa and IRb) of 27,172 bp in length. The repeat regions divided the genome into two single-copy regions, SSC and LSC with17,847 and 86,422 bp, respectively. The overall GC-content of the *T. fortunei* cp genome is 37.21%. It encodes a total of 109 unique genes, including 79 protein-coding genes, 26 *tRNA* genes, four *rRNA* genes. Twelve genes (rps16, atpF, rpoC2,…) contain a single intron, and 11 genes (trnk-UUU, trnS-CGA, ycf3,…) have two introns.

Phylogenetic analysis suggested that *T. fortunei* is closely clustered with *Chamaerops humilis* (Figure 1), which was generated based on the 24 complete cp genomes. The sequences were initially aligned using MAFFT (Katoh and Standley 2013). The phylogenetic tree was built using IQ-TREE (Nguyen et al. 2015) with 1000 bootstrap. The result shows a foundation for chloroplast genome engineering of *T. fortunei* in the future.

**Figure 1.**
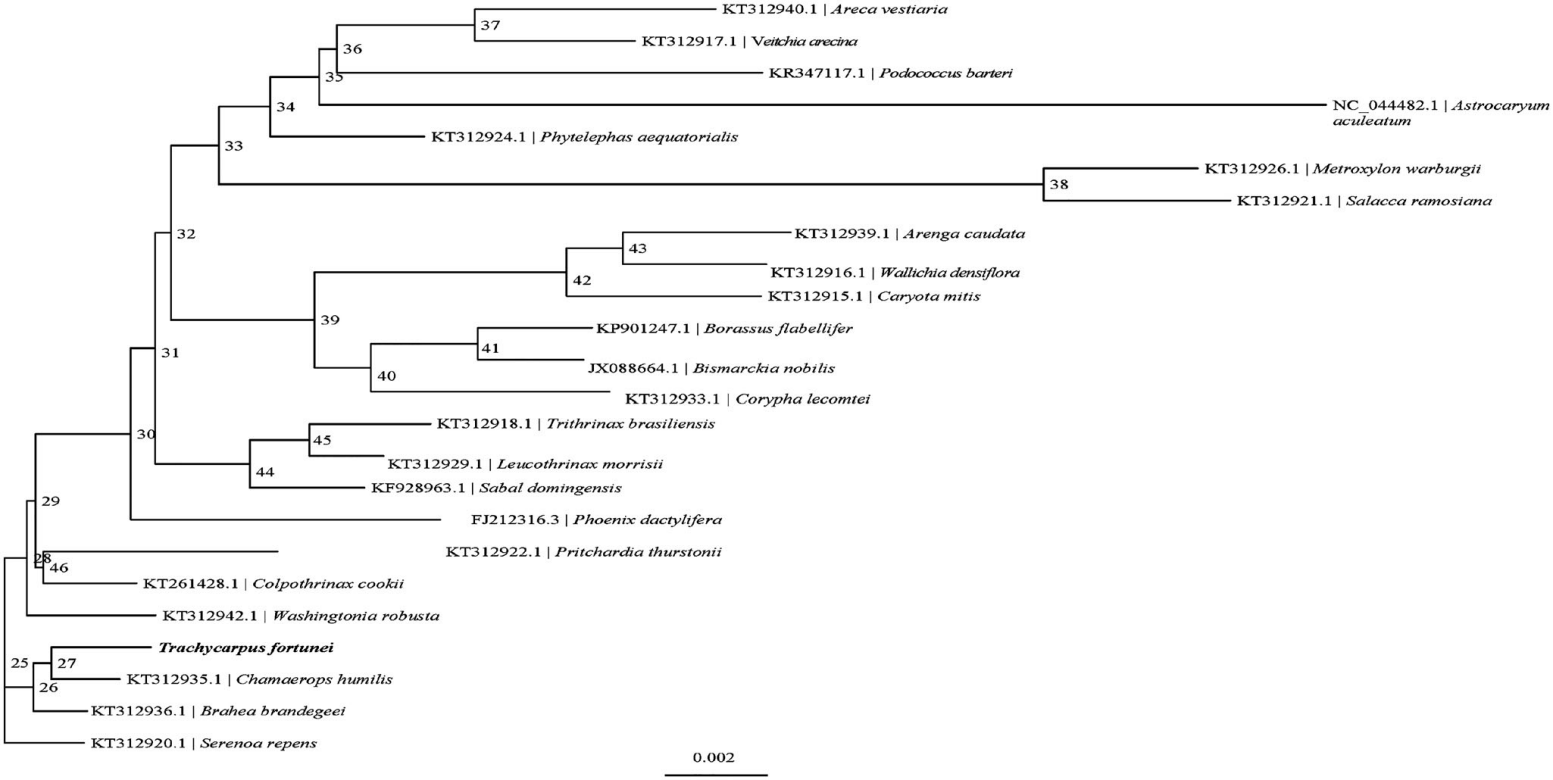
Phylogenetic relationships of 24 species based on the maximum-likelihood (ML) analysis of *T. fortunei.*

## Data Availability

The data that support the findings of this study are openly available in GenBank of NCBI at http://www.ncbi.nim.nih.gov, reference number MT712077.
